# Occupational Safety and Health among Brick Workers in the Old Testament (*Pentateuch*)

**DOI:** 10.5334/aogh.2493

**Published:** 2019-05-03

**Authors:** Elias E. Mazokopakis

**Affiliations:** 1 Department of Internal Medicine, Naval Hospital of Crete, Chania, GR; 2 Department of Theology, School of Theology, National and Kapodistrian University of Athens, Athens, GR

Dear Editor,

I read with great interest the article by Rupakheti et al. [1] about the occupational safety and health vulnerability among brick factory workers in Dhading District, Nepal. However, a story of occupational safety and health among brick workers has been described in the Bible and particularly in the Book of Exodus, the second book of the *Pentateuch* or *Torah*, and occurred probably during the 13th century BC. It is known that the Egyptians oppressed the Israelites in Egypt with the hard labor of brick construction. According to the biblical text, when a new pharaoh (18th or 19th Egyptian Dynasty; probably Ramses II; reign: 1279–1213 BC, 19th Egyptian Dynasty), who did not know Joseph, came to power in Egypt, fearing that the Israelites might join their enemies, fight against Egyptians, and depart from the land, he “appointed taskmasters over them to afflict them with hard labor. And they built for Pharaoh storage cities, Pithom and Raamses…The Egyptians compelled the sons of Israel to labor rigorously; and they made their lives bitter with hard labor in mortar and bricks and at all kinds of labor in the field, all their labors which they rigorously imposed on them” (Exodus 1: 8–14). The forced labor imposed on the Israelites is portrayed in a mural of ancient Thebes (Theban Tomb 100), which depicted slaves manufacturing and transporting bricks (see Figures 1 and 1a). The Israelites’ status in Egypt was made more difficult when they had to gather their own straw and still produce the same number of bricks (Exodus 5: 7–19). We point out that manufacturing bricks was laborious work that included removing foreign substances from the mud or clay and mixing it with chopped straw or other plant material as a stabilizer. The addition of straw to the clay increased the durability of the bricks produced. The mixture of mud or clay and straw was moistened with water, trampled underfoot, and then molded by hand or pressed into a four-sided wooden “brick mold” (Nahum 3: 14). The sides of the molds were probably dusted with dry earth so that the molds could be slipped off easily. Often, while the brick was still wet, it was stamped with the mark of the reigning monarch. The bricks were then left to dry in the sun or were kiln dried. Kiln-dried bricks were superior in quality to sun-dried bricks. The latter tended to disintegrate when subjected to floods and shrivel under the intense heat of the summer sun.

**Figure 1:**
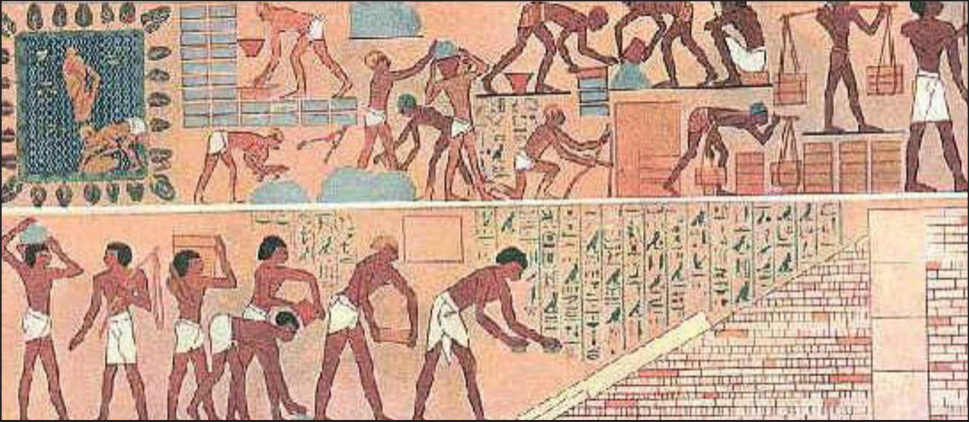
Slaves (probably Israelites) manufacture and transport bricks in Egypt. Mural from the private tomb (Theban Tomb 100; TT100) of vizier (during the reigns of Thutmosis III and Amenhotep II, 18th Egyptian Dynasty) Rekhmire on the West Bank at Luxor (ancient Thebes). At the left, water is being town to moisten the clay. Next clay is kneaded and then (center) carried to two men who are making bricks into molds. Finally, the bricks are put out to dry. In the lower panel, the temple is being built of the new bricks.

**Figure 1a:**
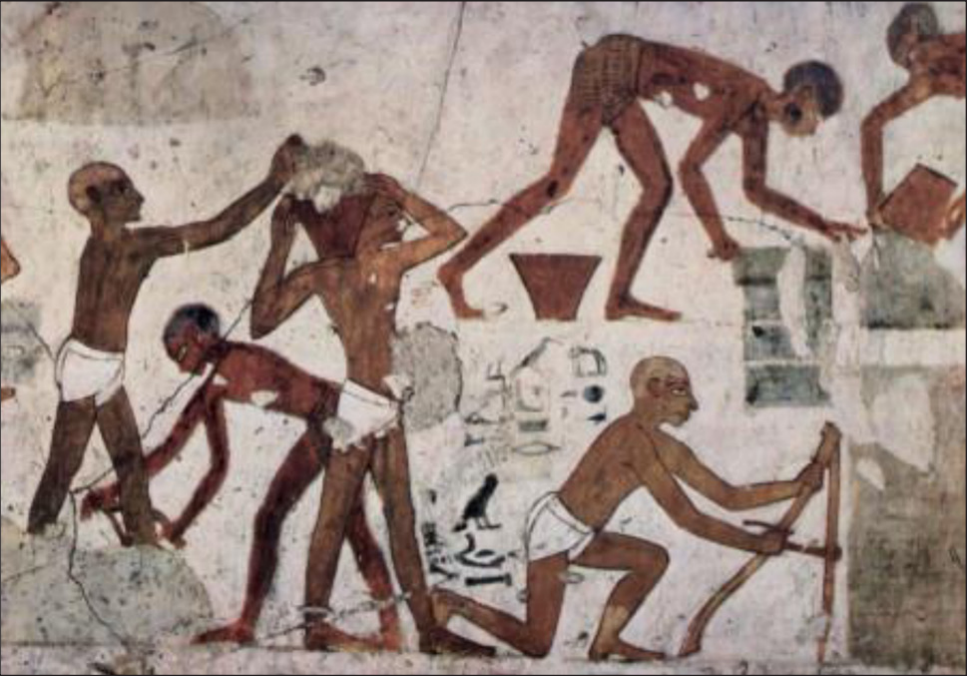
Detail of Figure 1.

When at work, the Israelites experienced not only physical stress, but also mental (emotional) stress. The allocation and overload of heavier work after Moses and Aaron’s first encounter with the pharaoh (19th Egyptian Dynasty; probably Merneptah; reign: 1213–1203 BC) (Exodus 5: 6–9), the underestimation of complaints expressed (Exodus 5: 17–18), the lack of recognition and the feeling of injustice (Exodus 5: 8, 15–18) as well as the enforced hostile work environment (Exodus 5: 5, 13–14) were some of the causes of work-related stress among the Israelites. As it is known, work-related stress is a set of harmful reactions, physical and emotional, that occurs when there is no balance between the requirements of the workplace and the capabilities, resources, or needs of the worker. According to the biblical text, the Israelites did not listen to the three promises of the Lord from Moses (Exodus 6: 6–8) “on account of their spirit despondency and cruel bondage” (Exodus 6: 9). This phrase indicates the role of work-related stress as a determinant of depression which medical literature confirms [2,3,4,5]. This biblical story of the work-related oppression suffered by the Israelites in Egypt is one of the medical reports of work-related stress in the history of occupational medicine. We draw attention to the harsh working conditions and work-related health problems (e.g. accidents, injuries, snake bites, and scorpion stings) faced by the Egyptian workers (and not Israelite slaves) during the construction of the pyramids centuries ago in the biblical story of Israelite slaves in Egypt, as described in *Histories* (Book II: Euterpe, 124 & 125) by the famous Greek historian Herodotus of Halicarnassus (fifth century BC) and in the ancient Egyptian medical papyri (such as Ebers and Edwin Smith papyri) [6,7]. Moreover, the Old Testament addresses other issues of occupational safety and health, such as human injuries from farm animals (Exodus 21: 28–32) and the treatment of slaves (Exodus 21: 1–11; Deuteronomy 15: 12–18).
